# Recent Advances in Anti-Angiogenic Therapy of Cancer

**DOI:** 10.18632/oncotarget.234

**Published:** 2011-03-07

**Authors:** Rajeev S. Samant, Lalita A. Shevde

**Affiliations:** Mitchell Cancer Institute, University of South Alabama, Mobile, AL, USA

**Keywords:** angiogenesis, tumor progression, metastasis, VEGF, angiogenesis inhibitors

## Abstract

Since angiogenesis is critical for tumor growth and metastasis, anti-angiogenic treatment is a highly promising therapeutic approach. Thus, for over last couple of decades, there has been a robust activity aimed towards the discovery of angiogenesis inhibitors. More than forty anti-angiogenic drugs are being tested in clinical trials all over the world. This review discusses agents that have approved by the FDA and are currently in use for treating patients either as single-agents or in combination with other chemotherapeutic agents.

## WHAT IS ANGIOGENESIS?

The process of formation of new blood vessels from pre-existing blood vessels is termed as angiogenesis. In normal physiology, angiogenesis is necessary for repair and healing of tissues. Classically, there are sub-types of angiogenesis e.g., Sprouting angiogenesis which involves stimulation of endothelial cells to proliferate into the surrounding matrix and form solid sprouts extending towards the angiogenic stimulus leading to the formation of an entirely new vessel [1, 2]; Intussusception or splitting angiogenesis which involves division of the lumen of an existing vessel resulting in formation two vessels [3, 4]; Vasculogenesis, which is the formation of vasculature from endothelial stem cells or angioblasts, which proliferate into de-novo endothelial cells [5].

However, from the cancer biology perspective, angiogenesis is one of the most critical steps in the hematogenous metastasis as it provides the escape route for the tumor cells from the confines of the primary tumor and allows their seeding in distant organs [6].

## TUMOR ANGIOGENESIS

Some of the early classic experiments have demonstrated that tumor angiogenesis is indispensable for the growth of solid tumors [7, 8]. Tumor angiogenesis is generation of a network of blood vessels within the cancerous growth. This process can occur two ways: The more accepted model involves the release of signaling molecules by the tumor cells; these molecules activate the surrounding tissue to promote growth of new blood vessels. This stimulates vascular endothelial cells to divide rapidly [9, 10]. The other model proposes the generation of new vasculature by vasculogenic mimicry. This model argues that the tumor cells trans-differentiate in endothelial-like cells and create structures from inside of the tumor tapping into a nearby blood vessel [4].

## ANGIOGENESIS AND METASTASIS

Escape of the tumor cell from the confines of the primary tumor to distant body parts is the pre-requisite for hematogenous metastasis. This escape route is provided by the tumor vasculature. Thus, it was envisioned that inhibition of angiogenesis will also lead to inhibition of metastasis. This phenomenon was demonstrated by very elegant mouse model studies using angiostatin [11, 12]. Angiostatin was also demonstrated to be secreted by some primary tumors leading to restricted growth of the metastasis leading to “dormancy” of the metastasis. Mice deficient in angiogenesis (Id1 & Id3 deficient) showed significantly less tumor take rates [13]. Independent studies showed absence of metastasis in angiogenesis deficient mice [14, 15]. Defective angiogenesis was attributed to impaired VEGF-dependent recruitment of precursor endothelial cells from the bone marrow to the newly developing tumor vasculature [16].

## LYMPHANGIOGENESIS

Metastasis of malignant tumors to regional lymph nodes is one of the early signs of cancer spread in patients, and it occurs at least as frequently as hematogenous metastasis [17]. Particularly, in cancers, such as breast cancer, lymphatic metastasis is a predominant route for tumor spread. The contribution of lymphatic system to the tumor growth is an area that is relatively less studied. However, lymphatic vessels are speculated to contribute to tumor growth and metastasis in a variety of ways. The VEGF, FGF2 and PDGF produced by vascular endothelial cells are proposed to be involved in the activation of lymphatic endothelial cells, which in turn produce matrix metalloproteases and urokinase plasminogen activator (uPA) that can promote malignant tumor growth. Thus, there exists a synergistic crosstalk between the tumor and the lymphatic vessels and blood vessels.

## REGULATORS OF TUMOR ANGIOGENESIS

Angiogenesis is a complex and intricately regulated process. Like all other regulated biological phenomena, angiogenesis has activators or pro-angiogenic factors and inhibitors or anti-angiogenic factors [9].

### The Activators

Tumor cells activate signaling pathways that promote uncontrolled proliferation and survival. These include the PI3K/AKT/mTOR pathway, Hedgehog pathway and, Wnt pathway [18-24] that produce pro-angiogenic signaling intermediates [25, 26]. Among the several reported activators of angiogenesis present in cells two proteins appear to be the most important for sustaining tumor growth: vascular endothelial growth factor (*VEGF*) and basic fibroblast growth factor (*bFGF*). VEGF and bFGF are secreted by the tumor into the surrounding tissue. They bind to their cognate receptors on endothelial cells. This activates a signaling cascade that transmits a nuclear signal prompting target genes to activate endothelial cell growth. Activated endothelial cells also produce matrix metalloproteinases (MMPs). These MMPs break down the extracellular matrix and allow the migration of endothelial cells. The division and migration of the endothelial cells leads to formation of new blood vessels [27, 28].

### The Inhibitors

If angiogenesis is so critical for the tumor growth, then agents that inhibit angiogenesis would have great therapeutic value. With the discovery of endostatin, the concept of anti-angiogenic therapy was launched and popularized by Dr. Folkman [29]. Angiogenesis inhibitors have been discovered from a variety of sources. Some are naturally present in the human body e.g. specific fragments of structural proteins such as collagen or plasminogen (angiostatin, endostatin, tumstatin) [30]. Others are natural products in green tea, soy beans, fungi, mushrooms, tree bark, shark tissues, snake venom etc. [31]. A plethora of synthetic compounds are also characterized to have anti-angiogenic properties [32].

## ANTI-ANGIOGENIC TREATEMENT OF CANCER

**Figure 1:**
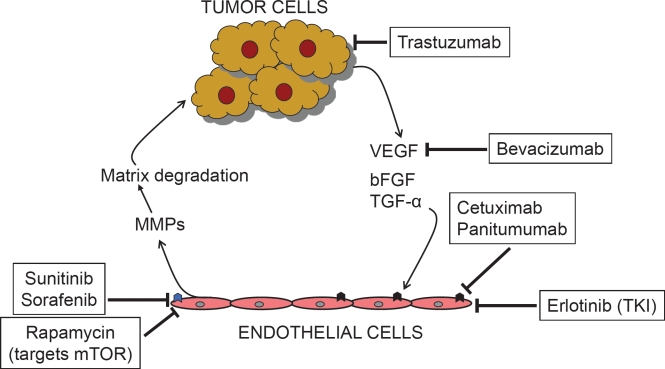
Targets of FDA-approved angiogenesis inhibitors: Angiogenesis inhibitors impact both, the tumor as well as the endothelial cells resulting in the disruption of the effects of the microenvironment in promoting tumor growth and angiogenesis Tumor cells produce pro-angiogenic agents including VEGF, bFGF and TGF-α by the signaling pathways such as Hh pathway (19) as well as their intermediates including oncoproteins such as COX-2 and osteopontin (25, 26). These factors signal and activate endothelial cells that produce proteases such as MMPs that facilitate invasive properties of the tumor cells. Bevacizumab binds to and squelches the availability of VEGF. Cetuximab and Panitumumab inhibit the activities of VEGF receptor (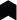). Tumor cells themselves are negatively impacted by Trastuzumab that deprives the effects of HER-2. Small molecule tyrosine kinase inhibitors such as Erlotinib, Sorafenib and Sunitinib block the activity of multiple growth factor receptors (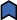) including VEGF receptors, PDGF-receptors, RET and Raf-1. Rapamycin targets mTOR. The exact molecular targets of other agents including thalidomide and Bortezomib are not fully understood.

Since angiogenesis is an event critical to primary tumor growth as well as metastasis, anti-angiogenic treatment of tumors is a highly promising therapeutic avenue [33]. Thus, for over last couple of decades, there has been a robust activity aimed towards the discovery of angiogenesis inhibitors [34, 35]. More than forty anti-angiogenic drugs are being tested in human cancer patients in clinical trials all over the world. From the several anti-angiogenic agents reported, we have focused this review on discussing those agents that have received FDA approval in the United States and are currently in use for treating patients either as a single-agent or in combination with other chemotherapeutic agents (Figure 1). Based on functionality, the anti-angiogenic drugs can be sub-divided into three main groups:

### Drugs that inhibit growth of endothelial cells

e.g. Endostatin and combretastatin A4, cause apoptosis of the endothelial cells [36]. Thalidomide is also a potent inhibitor of endothelial cell growth [37].

### Drugs that block angiogenesis signaling

e.g. anti-VEGF antibodies (Avastin, FDA approved for colorectal cancer), Interferon-alpha (inhibits the production of bFGF and VEGF) [36].

### Drugs that block extracellular matrix breakdown

e.g. inhibitors of MMPs [38].

## ANTI-ANGIOGENIC THERAPIES THAT HAVE RECEIVED USA-FDA APPROVAL

Conventional chemotherapy is usually a systemic therapy that tries to capture a narrow therapeutic window offered by rapid proliferation of tumor cells compared to the normal cells. Chemotherapy has significant side effects such as hair loss, diarrhea, mouth ulcer, infection, and low blood counts. Anti-angiogenic therapy has several advantages over chemotherapy as it is mostly not directed towards directly killing cells but stopping the blood vessel formation, an event that is rare in tissues other than growing tumor. Hence it is well tolerated by the patients and has fewer side effects [29]. There are currently seven approved anti-cancer therapies in two primary categories:

1. Monoclonal antibodies directed against specific pro-angiogenic growth factors and/or their receptors
2. Small molecule tyrosine kinase inhibitors (TKIs) of multiple pro-angiogenic growth factor receptors.

Besides these, inhibitors of mTOR (mammalian target of rapamycin), proteasome inhibitors and thalidomide have also been reported to indirectly inhibit angiogenesis through mechanisms that are not completely understood.

## MONOCLONAL ANTIBODY THERAPIES

Four monoclonal antibody therapies are approved to treat several tumor types:

### Bevacizumab (Avastin®)

The first FDA approved angiogenesis inhibitor, Avastin is a humanized monoclonal antibody that binds biologically active forms of vascular endothelial growth factor (VEGF) and prevents its interaction with VEGF receptors (VEGFR-1 and VEGFR-2), thereby inhibiting endothelial cell proliferation and angiogenesis. Bevacizumab has been tested in phase I studies in combination with chemotherapy with a good safety profile [39]. This treatment is approved for metastatic colorectal cancer and non-small cell lung cancer [40-43]. Bevacizumab has also evolved as a first line of treatment in combination with paclitaxel in breast cancer patients by virtue of its ability to double median progression-free survival (PFS) [44]. In combination with chemoendocrine therapy (including capecitabine and vinorelbine, and letrozole) bevacizumab treatment significantly decreased the percentage of viable circulating endothelial cells and prevented the chemotherapy-induced mobilization of circulating progenitors [45]. In combination with irinotecan, bevacizumab significantly increased PFS in glioma patients [46, 47]. VEGF has emerged as a compelling therapeutic target for leukemias. Inhibition of angiogenesis in hematological malignancies interdicts the angiogenesis within the bone marrow ecosystem comprised of multiple cell types, including fibroblasts, endothelial progenitor cells, endothelial cells, dendritic cells and, malignant cells, blocking the availability of nutrients to cancer cells and disrupting crosstalk between the various cell types to curtail the malignant phenotype [48].

### Cetuximab (Erbitux®)

This is a monoclonal antibody that binds the extracellular domain of epidermal growth factor receptor (EGFR), preventing ligand binding and activation of the receptor resulting in internalization and degradation of the receptor culminating in inhibition of cell proliferation and angiogenesis. Cetuximab downregulated VEGF expression in a dose-dependent manner in a human colorectal carcinoma (CRC) cell line and in human CRC mouse xenografts [49]. The xenografts also showed a significant reduction in blood vessel counts following several rounds of cetuximab treatment [49], indicating that the tumor-promoting effects of EGFR overexpression may be mediated through VEGF stimulation and tumor angiogenesis. This treatment is approved for metastatic CRC and head and neck cancer [50] in patients who are refractory to irinotecan-based chemotherapy. In combination with irinotecan (an inhibitor of topoisomerase I), cetuximab is the first monoclonal antibody that has been approved by the FDA as second-line treatment for metastatic colorectal cancer [51, 52]. In Phase I and Phase III trials [53, 54] cetuximab significantly improved the effects of radiotherapy in patients with unresectable (cannot be removed by surgery) squamous cell carcinoma of the head and neck (SCCHN). Cetuximab has also been shown to sensitize cells to radiation and chemotherapy, potentially through blocking EGFR nuclear import and the associated activation of DNA protein kinase enzymes necessary for repairing radiation- and chemotherapy-induced DNA damage [55]. Compared to radiation alone, cetuximab plus radiation therapy can nearly double the median survival in patients with a certain kind of head and neck cancer that has not spread to other parts of the body [54] making cetuximab the only drug achieving interesting response rate in second line treatment of advanced SCCHN [56]. Cetuximab was also found to be tolerated well in combination with cisplatin, or carboplatin, and fluorouracil [57, 58].

### Panitumumab (Vectibix™)

It is a fully humanized anti-EGFR monoclonal antibody that binds specifically to the human EGFR. Panitumumab is a recombinant human monoclonal antibody [59]; therefore, the risk of an infusion reaction is minimized. Vectibix® is indicated as a single agent for the treatment of EGFR-expressing, metastatic colorectal carcinoma with disease progression on or following fluoropyrimidine-, oxaliplatin-, and irinotecan-containing chemotherapy regimens [60-62]. The effectiveness of Vectibix® as a single agent for the treatment of EGFR-expressing, metastatic CRC is based on progression-free survival [63, 64]. Panitumumab is used in patients who are not responding to regimens containing fluorouracil, oxaliplatin, and irinotecan [60]. Patients often receive panitumumab after receiving bevacizumab or cetuximab. Panitumumab can be given with FOLFOX (oxaliplatin, leucovorin, and fluorouracil) or FOLFIRI (irinotecan, leucovorin, and fluorouracil) regimens, or as a single agent. Currently no data are available that demonstrate an improvement in disease-related symptoms or increased survival with Vectibix® in colon cancer [65]. This drug is also being tested for aerodigestive track and head and neck cancer [66, 67].

### Trastuzumab (Herceptin®)

Is a humanized monoclonal antibody that binds the extracellular domain of HER-2, which is overexpressed in 25-30% of invasive breast cancer tumors [68]. HER2-positive breast cancer is highly aggressive disease with high recurrence rate, poorer prognosis with decreased survival compared with HER2-negative breast cancer [69]. Herceptin® is designed to target and block the function of HER2 protein overexpression. This is the first humanized antibody is approved for Breast cancer [70]. Herceptin® is approved by the FDA to treat HER2 positive breast cancer that has metastasized after treatment with other anticancer drugs [71]. It is also approved to be used with other drugs to treat HER2-positive breast cancer that has spread to the lymph nodes to be used after surgery. The FDA first approved Herceptin in September 1998 [71-73]. In November 2006, the FDA approved Herceptin as part of a treatment regimen containing doxorubicin, cyclophosphamide and paclitaxel, for the adjuvant treatment of patients with HER2-positive, node-positive breast cancer (http://www.fda.gov/NewsEvents/Newsroom/PressAnnouncements/default.htm). In January 2008, the FDA approved Herceptin as a single agent for the adjuvant treatment of HER2-overexpressing node-negative (ER/PR-negative or with one high-risk feature) or node-positive breast cancer, following multi-modality anthracycline-based therapy (http://biopharminternational.findpharma.com/biopharm/News/FDA-Approves-Expanded-Adjuvant-Indications-for-Her/ArticleStandard/Article/detail/518867). Trastuzumab is also being studied in the treatment of other types of cancers such as pancreatic [74], endometrial [75], lung [76], cervical [77] and ovarian cancer [78]

### SMALL MOLECULE TYROSINE KINASE INHIBITORS (TKIs)

Protein tyrosine kinases have emerged as crucial targets for therapeutic intervention in cancer especially because they play an important role in the modulation of growth factor signaling. As per ClinicalTrials.gov (www.clinicaltrials.gov), there are 43 ongoing studies on tyrosine kinase inhibitors in angiogenesis. Since discussing all of them is beyond the scope of this article, we have focused our discussion on the three TKIs that are currently approved as anti-cancer therapies:

#### Erlotinib (Tarceva®)

Erlotinib hydrochloride (originally coded as OSI-774) is an orally available, potent, reversible, and selective inhibitor of the EGFR (ErbB1) tyrosine kinase activity. Erlotinib hydrochloride has been approved by FDA for treatment of patients with locally advanced or metastatic NSCLC after failure of at least one prior chemotherapy regimen [79, 80]. Interesting recent studies have demonstrated that since Erlotinib and Bevacizumab act on two different pathways critical to tumor growth and dissemination, administering these drugs concomitantly may confer additional clinical benefits to cancer patients with advanced disease. This combination therapy may prove to be a viable second-line alternative to chemotherapy in patients with NSCLC [81]. Also, for patients with locally advanced, unresectable or metastatic pancreatic carcinoma, Erlotinib has received FDA approval for the treatment in combination with gemcitabine [82, 83]. Erlotinib is also being studied in the treatment of other types of cancers. For example combination of Erlotinib with Bevacizumab has been evaluated in metastatic breast cancer [84], hepatocellular carcinoma [85] and in metastatic renal cancer [86] as phase II trials. Outcomes for prostate, cervical and colorectal cancers treated with Erlotinib are cautiously optimistic [87-89].

#### Sorafenib (Nexavar®)

Sorafenib is an orally active inhibitor of VEGFR-1, VEGFR-2, VEGFR-3, PDGFR-β, and Raf-1 tyrosine kinase activity [90]. It has received the approval of FDA for the treatment of patients with unresectable hepatocellular carcinoma [91] and advanced renal cell carcinoma [92]. However, not all advanced hepatocellular carcinoma patients were able to tolerate sorafenib and some patients experienced tumor progression [91]. Sorafenib has shown improvements in PFS in patients with renal cell carcinoma [93]. It is one of the aggressively studied drugs. According to the NCI clinical trials search results, there are about 168 active clinical trials involving sorafenib in a variety of cancers.

#### Sunitinib (Sutent®)

Sunitinib targets activity of multiple tyrosine kinases such as VEGFR-1, VEGFR-2, VEGFR-3, PDGFR- β, and RET [94]. It is approved by FDA as Sunitinib malate for treating advanced (metastatic) renal cell carcinoma [95]. It is also approved by FDA for gastrointestinal stromal tumor (GIST) in patients whose disease has progressed or who are unable to tolerate treatment with imatinib (Gleevec), the current treatment for GIST patients [95, 96]. Sunitinib has shown early evidence of anti-tumor activity in Phase II trials in US, European and Asian patients with locally advanced, unresectable and metastatic hepatocellular carcinoma. A Phase III trial of sunitinib in hepatocellular carcinoma is ongoing [97]. According to the NCI clinical trials search results, Sunitinib is currently evaluated in about 150 active clinical trials. It is evaluated for ovarian [98], breast [99] and non small cell lung cancer [100] among others [101].

#### Inhibitors of mTOR

mTOR plays a part in the PI3 kinase/AKT pathway involved in tumor cell proliferation and angiogenesis [102]. Rapamycin and related mTOR inhibitors inhibit endothelial cell VEGF expression, as well as VEGF-induced endothelial cell proliferation [103]. Inhibitors of mTOR are an important class of anti-angiogenic agents. These include: deforolimus, everolimus, rapamycin (sirolimus), and temsirolimus [104, 105]. Temsirolimus (Toricel™) is a small molecule inhibitor of mTOR, approved for treating advanced renal cell carcinoma [106]. It is a type of rapamycin analog and a type of serine/threonine kinase inhibitor, it is also called CCI-779. In pre-clinical models combination therapy for treating breast cancer using anti-estrogen, ERA-923, and temsirolimus has been successfully tested [107]. It is found to be highly effective against human melanoma when tested in combination with cisplatin and DTIC (in independent studies) in a SCID mouse xenotranplantation model [108, 109]. There are over 41 active studies of Temsirolimus for a variety of solid tumors [110]. mTOR inhibition has also been strongly advocated in as a putative cancer therapeutic strategy for urologic malignancies [111]. In a pilot study (6 patients) with imatinib-resistant CML, rapamycin induced major and minor leukocyte responses, with an observed decrease in the mRNA levels of VEGFA in circulating leukaemic cells [112]. Combination treatments for breast cancer with aromatase inhibitor [113] and letrozol [114] are also being evaluated. Rapamycin treatment brought partial responses (>50% reduction in the absolute number of blood blasts) and stable disease in adult refractory/relapsed AML [115]. In a recent report, Deforolimus was studied in a Phase 2 trial in pretreated patients with various hematological malignancies, including ALL, AML, CLL, CML, MDS, agnogenic myeloid metaplasia, mantle cell lymphoma and T-cell leukemia/lymphoma [116]. Overall, 40% of deforolimus-treated patients experienced hematological improvement or stable disease.

### OTHER ANGIOGENIC AGENTS

#### Bortezomib (Velcade®)

Is a proteasome inhibitor that disrupts signaling of cancer cells, leading to cell death and tumor regression. It is the first compound in its class to be used in clinical practice. It has indirect anti-angiogenic properties [117]. While its exact mechanism is not understood, it induces the pro-apoptotic BH3-only family member NOXA in a p53 independent fashion triggering of a caspase cascade culminating in apoptosis in melanoma and myeloma cells [118]. It is FDA-approved for the treatment of myeloma that has relapsed after two prior treatments (or where resistance has developed following the last treatment). It was also found to induce high quality responses as third line salvage therapy with acceptable toxicity in a significant proportion of homogeneously pre-treated myeloma patients with progressive disease after autologous transplantation and thalidomide. [119]. In a Phase 3 trial involving 669 myeloma patients treated with at least one prior therapy, bortezomib increased median, improved overall survival, and increased response rate, compared with high-dose dexamethasone [120]. In combination with doxorubicin and gemcitabine, bortezomib was also found to be effective in heavily pretreated, advanced Cutaneous T cell Lymphomas (CTCL) [121]. Bortezomib was also reported to be active as a single agent for patients with relapsed/refractory CTCL and Peripheral T Cell Lymphoma (PTCL) with skin involvement [122]. On the contrary, the use of bortezomib was discouraged after a phase II study revealed that found in combination with dexamethasone, bortezomib is not active in heavily pre-treated patients with relapsed Hodgkin's lymphoma [123, 124].

#### Thalidomide (Thalomid®)

Possesses immunomodulatory, anti-inflammatory, and anti-angiogenic properties, although the precise mechanisms of action are not fully understood. Thalidomide was the first angiogenesis inhibitor to demonstrate clinical efficacy in multiple myeloma [37, 125]. Specifically in myeloma, thalidomide down-regulated VEGF secretion from bone marrow endothelial cells obtained from patients with active disease. In a landmark Phase 2 clinical trial, 169 previously treated patients with refractory myeloma received thalidomide monotherapy [126]. Partial response, was achieved in 30% of patients, and 14% achieved a complete or nearly complete remission. The survival rate at 2 years was 48%. These results led to many subsequent clinical studies of thalidomide in myeloma, leading ultimately to FDA approval of the drug in 2006, for the treatment of newly diagnosed multiple myeloma, in combination with dexamethasone. In the pivotal Phase 3 trial, the response rate in patients receiving thalidomide plus dexamethasone was 63% compared to 41% with dexamethasone alone [127]. Long-term outcome measures, including time-to-progression (TTP) and PFS, were recently reported for a 470 patient randomized, placebo-controlled Phase 3 clinical trial of a similar protocol in newly diagnosed multiple myeloma, with comparable overall response rates [128]. Significant increases resulted in both median TTP and median PFS for the thalidomide plus dexamethasone group versus dexamethasone alone.

Thalidomide was found to be moderately tolerated and minimally effective in patients with histologically proven advanced hepatocellular carcinoma [129]. Thalidomide provided no survival benefit for patients with multiple, large, or midbrain metastases when combined with WBRT (whole-brain radiation therapy) [130]. On the contrary, thalidomide did not significantly add to the efficacy of the fludarabine, carboplatin, and topotecan (FCT) regimen in poor prognosis AML patients [131] and was also ineffective in improving prognosis or decreasing plasma VEGF levels in patients with persistent or recurrent leiomyosarcoma of the uterus [132].

### METRONOMIC THERAPY

While conventional anti-angiogenic therapy is based on Maximum Tolerated Doses (MTD), the cells involved in angiogenesis may regenerate during the three- to four-week interval between cycles of the chemotherapy. Taking advantage of the fact that endothelial cells are about 10–100 times more susceptible to chemotherapeutic agents than cancer cells, therapy based on daily, oral, low-dose chemotherapeutic drugs was designed. Metronomic chemotherapy refers to the close, rhythmic administration of low doses of cytotoxic drugs, with minimal or no drug-free breaks, over prolonged periods. Metronomic therapy appears promising mainly due to the fact that its anti-angiogenic and anti-tumorigenic effects are accompanied by low toxicity, limited side effects, no need for hospitalization and allowing for feasible combinations with selective inhibitors of angiogenesis. There are several foreseeable advantages and opportunities for metronomic chemotherapy: activity against the parenchymal and stromal components, pro-apoptotic activity, reduction of the likelihood of emergence of acquired resistance, feasibility of long term administration and acceptable systemic side effects [133]. In a pilot phase II study conducted by Correale *et al* [134] to investigate the toxicity and activity of the novel metronomic regimen of weekly cisplatin and oral etoposide in high-risk patients with NSCLC, the objective response rate was 45.2%, disease control was 58.1%, meantime to progression and survival were 9 and 13 months, respectively. Pharmacokinetic analysis showed that this regimen allowed a greater median monthly area under the curve of the drugs than conventional schedules. In a Phase I trial of metronomic dosing of docetaxel and thalidomide, of the 26 patients with advanced tumors enrolled, prolonged freedom from disease progression was observed in 44.4% of the evaluable patients [135].

Circulating endothelial progenitor cells (EPCs) also participate in tumor angiogenesis. In a study comparing the effects of metronomic chemotherapy over conventional dose-dense chemotherapy, it was found that the numbers of circulating EPCs and the plasma levels of VEGF increased sharply, doubling pre-therapeutic levels at day 21 after conventional chemotherapy, whereas under low-dose metronomic chemotherapy, the numbers of circulating EPCs decreased significantly and VEGF plasma concentrations remained unchanged. These observations provide evidence that conventional dose-dense chemotherapy leads to rebound EPC mobilization even when given with adjuvant intention, while low-dose metronomic scheduling of cytotoxic substances such as trofosfamide may sharply reduce EPC release into the circulation. [136].

Combined bevacizumab and metronomic oral cyclophosphamide was also discovered to be a safe and effective regimen for heavily pre-treated ovarian cancer patients [137]. Treatment with metronomic capecitabine and cyclophosphamide in combination with bevacizumab was shown to be effective in advanced breast cancer and additionally was minimally toxic [138]. Metronomic treatment with carboplatin and vincristine associated with fluvastatin and thalidomide significantly increased survival of pediatric brain stem tumor patients. Tumor volume showed a significant reduction accompanied by increased quality of life [139]. Thus, given the fact that the most evident effect of selective anti-angiogenic agents (i.e. bevacizumab) is the significant prolonging of the duration of response obtainable by chemotherapy alone, with minimal possible side effects of cytotoxic agents given in association metronomic chemotherapy should be considered both as novel up-front or maintenance treatment in patients with biologically poorly aggressive advanced cancer diseases [140].

Overall, metronomic chemotherapy was able to induce tumor stabilization and prolong the duration of clinical benefit, without much associated toxicity. Emerging evidence suggests that metronomic chemotherapy could also activate the host immune system and potentially induce tumor dormancy [141-143].

### CONCLUSIONS AND FUTURE PERSPECTIVES

While angiogenesis as a hallmark of tumor development and metastasis is now a validated target for cancer treatment, the overall benefits of anti-angiogenic drugs from the perspective of impacting survival have left much to desire, endorsing a need for developing more effective therapeutic regimens e.g., combining anti-angiogenic drugs with established chemotherapeutic drugs [144, 145]. There are now several agents that target the tumor vasculature through different pathways, either by inhibiting formation of the tumor neovasculature or by directly targeting the mature tumor vessels. The main body of evolving evidence suggests that their effects are compounded by their synergistic use with conventional chemotherapy rather than individual agents. Anti-angiogenic drugs such as bevacizumab can bring about a transient functional normalization of the tumor vasculature. This can have an additive effect when co-administered with chemo/radiotherapy. But long term inhibition of angiogenesis reduces tumor uptake of co-administered chemotherapeutic agents. This underscores the need for discovering new targets for anti-angiogenic therapy in order to effectively prohibit angiogenesis and circumvent mechanisms that contribute to resistance mechanisms that emerge with long term use of anti-angiogenic therapies. It also warrants a need to define reliable surrogate indicators of effectiveness of the anti-angiogenic therapy as well as dependable markers for identifying the patients who are most likely to benefit from the combination of anti-angiogenic therapy and conventional chemotherapy.

Several new frontiers are emerging. New advances in understanding endothelial cells, which constitute the tumor vasculature, towards developing antiangiogenic strategies are one of the important ones [146, 147]. Novel cellular targets such as integrins and microRNAs and novel treatment options such as possible use of pharmaconutrients to modulate angiogenic pathways need careful testing and evaluation [148-151]. Finally, the administration of these drugs in a metronomic schedule is likely to improve the overall response to anti-angiogenic drugs making it feasible to administer them with conventionally toxic chemotherapeutic drugs, thus increasing the armamentarium of drug combinations that can be employed for treatment.

## References

[1] Risau W (1998). Development and differentiation of endothelium. Kidney Int Suppl.

[2] Risau W (1998). Angiogenesis is coming of age. Circ Res.

[3] Burri PH, Djonov V (2002). Intussusceptive angiogenesis--the alternative to capillary sprouting. Mol Aspects Med.

[4] Burri PH, Hlushchuk R, Djonov V (2004). Intussusceptive angiogenesis: Its emergence, its characteristics, and its significance. Dev Dyn.

[5] Patan S (2004). Vasculogenesis and angiogenesis. Cancer Treat Res.

[6] Weiss L, Orr FW, Honn KV (1989). Interactions between cancer cells and the microvasculature: A rate-regulator for metastasis. Clin Exp Metastasis.

[7] Shubik P (1982). Vascularization of tumors: A review. J Cancer Res Clin Oncol.

[8] Reinhold HS, van den Berg-Blok A (1984). Factors influencing the neovascularization of experimental tumours. Biorheology.

[9] Goh PP, Sze DM, Roufogalis BD (2007). Molecular and cellular regulators of cancer angiogenesis. Curr Cancer Drug Targets.

[10] Kos M, Dabrowski A (2002). Tumour's angiogenesis--the function of vegf and bfgf in colorectal cancer. Ann Univ Mariae Curie Sklodowska [Med].

[11] Folkman J (2002). Role of angiogenesis in tumor growth and metastasis. Semin Oncol.

[12] O'Reilly MS, Holmgren L, Shing Y, Chen C, Rosenthal RA, Moses M, Lane WS, Cao Y, Sage EH, Folkman J (1994). Angiostatin: A novel angiogenesis inhibitor that mediates the suppression of metastases by a lewis lung carcinoma. Cell.

[13] de Candia P, Solit DB, Giri D, Brogi E, Siegel PM, Olshen AB, Muller WJ, Rosen N, Benezra R (2003). Angiogenesis impairment in id-deficient mice cooperates with an hsp90 inhibitor to completely suppress her2/neu-dependent breast tumors. Proc Natl Acad Sci U S A.

[14] Fong S, Itahana Y, Sumida T, Singh J, Coppe JP, Liu Y, Richards PC, Bennington JL, Lee NM, Debs RJ, Desprez PY (2003). Id-1 as a molecular target in therapy for breast cancer cell invasion and metastasis. Proc Natl Acad Sci U S A.

[15] Gupta GP, Perk J, Acharyya S, de Candia P, Mittal V, Todorova-Manova K, Gerald WL, Brogi E, Benezra R, Massague J (2007). Id genes mediate tumor reinitiation during breast cancer lung metastasis. Proc Natl Acad Sci U S A.

[16] Lyden D, Hattori K, Dias S, Costa C, Blaikie P, Butros L, Chadburn A, Heissig B, Marks W, Witte L, Wu Y, Hicklin D, Zhu Z, Hackett NR, Crystal RG, Moore MA, Hajjar KA, Manova K, Benezra R, Rafii S (2001). Impaired recruitment of bone-marrow-derived endothelial and hematopoietic precursor cells blocks tumor angiogenesis and growth. Nat Med.

[17] Cao Y (2005). Opinion: Emerging mechanisms of tumour lymphangiogenesis and lymphatic metastasis. Nat Rev Cancer.

[18] Ching CB, Hansel DE Expanding therapeutic targets in bladder cancer: The pi3k/akt/mtor pathway. Lab Invest.

[19] Maun HR, Kirchhofer D, Lazarus RA Pseudo-active sites of protease domains: Hgf/met and sonic hedgehog signaling in cancer. Biol Chem.

[20] Das S, Harris LG, Metge BJ, Liu S, Riker AI, Samant RS, Shevde LA (2009). The hedgehog pathway transcription factor gli1 promotes malignant behavior of cancer cells by up-regulating osteopontin. J Biol Chem.

[21] Das S, Samant RS, Shevde LA Hedgehog signaling induced by breast cancer cells promotes osteoclastogenesis and osteolysis. J Biol Chem.

[22] Courtwright A, Siamakpour-Reihani S, Arbiser JL, Banet N, Hilliard E, Fried L, Livasy C, Ketelsen D, Nepal DB, Perou CM, Patterson C, Klauber-Demore N (2009). Secreted frizzle-related protein 2 stimulates angiogenesis via a calcineurin/nfat signaling pathway. Cancer Res.

[23] Fillmore RA, Mitra A, Xi Y, Ju J, Scammell J, Shevde LA, Samant RS (2009). Nmi (n-myc interactor) inhibits wnt/beta-catenin signaling and retards tumor growth. Int J Cancer.

[24] Mitra A, Menezes ME, Shevde LA, Samant RS Dnajb6 induces degradation of beta-catenin and causes partial reversal of mesenchymal phenotype. J Biol Chem.

[25] Ghosh N, Chaki R, Mandal V, Mandal SC Cox-2 as a target for cancer chemotherapy. Pharmacol Rep.

[26] Chakraborty G, Jain S, Kundu GC (2008). Osteopontin promotes vascular endothelial growth factor-dependent breast tumor growth and angiogenesis via autocrine and paracrine mechanisms. Cancer Res.

[27] Mignatti P, Rifkin DB (1996). Plasminogen activators and matrix metalloproteinases in angiogenesis. Enzyme Protein.

[28] Cierniewski CS, Malinowski M, Bednarek R, Cierniewska-Cieslak A (2007). Adhesive and proteolytic phenotype of migrating endothelial cells induced by thymosin beta-4. Ann N Y Acad Sci.

[29] Folkman J (2006). Antiangiogenesis in cancer therapy--endostatin and its mechanisms of action. Exp Cell Res.

[30] Folkman J (2004). Endogenous angiogenesis inhibitors. Apmis.

[31] Madhusudan S, Harris AL (2002). Drug inhibition of angiogenesis. Curr Opin Pharmacol.

[32] Yance DR, Sagar SM (2006). Targeting angiogenesis with integrative cancer therapies. Integr Cancer Ther.

[33] Fidler IJ, Ellis LM (1994). The implications of angiogenesis for the biology and therapy of cancer metastasis. Cell.

[34] Ruegg C, Mutter N (2007). Anti-angiogenic therapies in cancer: Achievements and open questions. Bull Cancer.

[35] Verhoef C, de Wilt JH, Verheul HM (2006). Angiogenesis inhibitors: Perspectives for medical, surgical and radiation oncology. Curr Pharm Des.

[36] Kerbel R, Folkman J (2002). Clinical translation of angiogenesis inhibitors. Nat Rev Cancer.

[37] D'Amato RJ, Loughnan MS, Flynn E, Folkman J (1994). Thalidomide is an inhibitor of angiogenesis. Proc Natl Acad Sci U S A.

[38] Chan LS, Daruwalla J, Christophi C (2008). Selective targeting of the tumour vasculature. ANZ J Surg.

[39] Herbst RS, Johnson DH, Mininberg E, Carbone DP, Henderson T, Kim ES, Blumenschein G, Lee JJ, Liu DD, Truong MT, Hong WK, Tran H, Tsao A, Xie D, Ramies DA, Mass R, Seshagiri S, Eberhard DA, Kelley SK, Sandler A (2005). Phase i/ii trial evaluating the anti-vascular endothelial growth factor monoclonal antibody bevacizumab in combination with the her-1/epidermal growth factor receptor tyrosine kinase inhibitor erlotinib for patients with recurrent non-small-cell lung cancer. J Clin Oncol.

[40] Herbst RS, O'Neill VJ, Fehrenbacher L, Belani CP, Bonomi PD, Hart L, Melnyk O, Ramies D, Lin M, Sandler A (2007). Phase ii study of efficacy and safety of bevacizumab in combination with chemotherapy or erlotinib compared with chemotherapy alone for treatment of recurrent or refractory non small-cell lung cancer. J Clin Oncol.

[41] Manegold C (2008). Bevacizumab for the treatment of advanced non-small-cell lung cancer. Expert Rev Anticancer Ther.

[42] Hochster HS (2006). Bevacizumab in combination with chemotherapy: First-line treatment of patients with metastatic colorectal cancer. Semin Oncol.

[43] Arkenau HT, Brunetto AT, Barriuso J, Olmos D, Eaton D, de Bono J, Judson I, Kaye S (2009). Clinical benefit of new targeted agents in phase i trials in patients with advanced colorectal cancer. Oncology.

[44] Sachdev JC, Jahanzeb M (2008). Evolution of bevacizumab-based therapy in the management of breast cancer. Clin Breast Cancer.

[45] Torrisi R, Bagnardi V, Cardillo A, Bertolini F, Scarano E, Orlando L, Mancuso P, Luini A, Calleri A, Viale G, Goldhirsch A, Colleoni M (2008). Preoperative bevacizumab combined with letrozole and chemotherapy in locally advanced er- and/or pgr-positive breast cancer: Clinical and biological activity. Br J Cancer.

[46] de Groot JF, Yung WK (2008). Bevacizumab and irinotecan in the treatment of recurrent malignant gliomas. Cancer J.

[47] Blagosklonny MV (2005). How avastin potentiates chemotherapeutic drugs: Action and reaction in antiangiogenic therapy. Cancer Biol Ther.

[48] Li WW, Hutnik M, Gehr G (2008). Antiangiogenesis in haematological malignancies. Br J Haematol.

[49] Petit AM, Rak J, Hung MC, Rockwell P, Goldstein N, Fendly B, Kerbel RS (1997). Neutralizing antibodies against epidermal growth factor and erbb-2/neu receptor tyrosine kinases down-regulate vascular endothelial growth factor production by tumor cells in vitro and in vivo: Angiogenic implications for signal transduction therapy of solid tumors. Am J Pathol.

[51] Vincenzi B, Santini D, Rabitti C, Coppola R, Beomonte Zobel B, Trodella L, Tonini G (2006). Cetuximab and irinotecan as third-line therapy in advanced colorectal cancer patients: A single centre phase ii trial. Br J Cancer.

[52] Gebbia V, Del Prete S, Borsellino N, Ferrau F, Tralongo P, Verderame F, Leonardi V, Capasso E, Maiello E, Bordonaro R, Stinco S, Agostara B, Barone C (2006). Efficacy and safety of cetuximab/irinotecan in chemotherapy-refractory metastatic colorectal adenocarcinomas: A clinical practice setting, multicenter experience. Clin Colorectal Cancer.

[53] Robert F, Ezekiel MP, Spencer SA, Meredith RF, Bonner JA, Khazaeli MB, Saleh MN, Carey D, LoBuglio AF, Wheeler RH, Cooper MR, Waksal HW (2001). Phase i study of anti--epidermal growth factor receptor antibody cetuximab in combination with radiation therapy in patients with advanced head and neck cancer. J Clin Oncol.

[54] Bonner JA, Harari PM, Giralt J, Azarnia N, Shin DM, Cohen RB, Jones CU, Sur R, Raben D, Jassem J, Ove R, Kies MS, Baselga J, Youssoufian H, Amellal N, Rowinsky EK, Ang KK (2006). Radiotherapy plus cetuximab for squamous-cell carcinoma of the head and neck. N Engl J Med.

[55] Dittmann K, Mayer C, Fehrenbacher B, Schaller M, Raju U, Milas L, Chen DJ, Kehlbach R, Rodemann HP (2005). Radiation-induced epidermal growth factor receptor nuclear import is linked to activation of DNA-dependent protein kinase. J Biol Chem.

[56] Merlano M, Occelli M (2007). Review of cetuximab in the treatment of squamous cell carcinoma of the head and neck. Ther Clin Risk Manag.

[57] Bourhis J, Rivera F, Mesia R, Awada A, Geoffrois L, Borel C, Humblet Y, Lopez-Pousa A, Hitt R, Vega Villegas ME, Duck L, Rosine D, Amellal N, Schueler A, Harstrick A (2006). Phase i/ii study of cetuximab in combination with cisplatin or carboplatin and fluorouracil in patients with recurrent or metastatic squamous cell carcinoma of the head and neck. J Clin Oncol.

[58] Baselga J, Pfister D, Cooper MR, Cohen R, Burtness B, Bos M, D'Andrea G, Seidman A, Norton L, Gunnett K, Falcey J, Anderson V, Waksal H, Mendelsohn J (2000). Phase i studies of anti-epidermal growth factor receptor chimeric antibody c225 alone and in combination with cisplatin. J Clin Oncol.

[59] Winkeljohn DL (2008). Review of panitumumab: A targeted therapy. Clin J Oncol Nurs.

[60] Giusti RM, Shastri KA, Cohen MH, Keegan P, Pazdur R (2007). Fda drug approval summary: Panitumumab (vectibix). Oncologist.

[61] Giusti RM, Shastri K, Pilaro AM, Fuchs C, Cordoba-Rodriguez R, Koti K, Rothmann M, Men AY, Zhao H, Hughes M, Keegan P, Weiss KD, Pazdur R (2008). U.S. Food and drug administration approval: Panitumumab for epidermal growth factor receptor-expressing metastatic colorectal carcinoma with progression following fluoropyrimidine-, oxaliplatin-, and irinotecan-containing chemotherapy regimens. Clin Cancer Res.

[62] Berlin J, Posey J, Tchekmedyian S, Hu E, Chan D, Malik I, Yang L, Amado RG, Hecht JR (2007). Panitumumab with irinotecan/leucovorin/5-fluorouracil for first-line treatment of metastatic colorectal cancer. Clin Colorectal Cancer.

[63] Hecht JR, Patnaik A, Berlin J, Venook A, Malik I, Tchekmedyian S, Navale L, Amado RG, Meropol NJ (2007). Panitumumab monotherapy in patients with previously treated metastatic colorectal cancer. Cancer.

[64] Van Cutsem E, Peeters M, Siena S, Humblet Y, Hendlisz A, Neyns B, Canon JL, Van Laethem JL, Maurel J, Richardson G, Wolf M, Amado RG (2007). Open-label phase iii trial of panitumumab plus best supportive care compared with best supportive care alone in patients with chemotherapy-refractory metastatic colorectal cancer. J Clin Oncol.

[65] Wainberg Z, Hecht JR (2006). Panitumumab in colon cancer: A review and summary of ongoing trials. Expert Opin Biol Ther.

[66] Brake R, Starnes C, Lu J, Chen D, Yang S, Radinsky R, Borges L (2008). Effects of palifermin on antitumor activity of chemotherapeutic and biological agents in human head and neck and colorectal carcinoma xenograft models. Mol Cancer Res.

[67] Kruser TJ, Armstrong EA, Ghia AJ, Huang S, Wheeler DL, Radinsky R, Freeman DJ, Harari PM (2008). Augmentation of radiation response by panitumumab in models of upper aerodigestive tract cancer. Int J Radiat Oncol Biol Phys.

[68] Nichols DW, Wolff DJ, Self S, Metcalf JS, Jacobs D, Kneuper-Hall R, Cate JCt (2002). A testing algorithm for determination of her2 status in patients with breast cancer. Ann Clin Lab Sci.

[69] Dean-Colomb W, Esteva FJ (2008). Her2-positive breast cancer: Herceptin and beyond. Eur J Cancer.

[70] Nahta R, Esteva FJ (2007). Trastuzumab: Triumphs and tribulations. Oncogene.

[71] Graziano C (1998). Her-2 breast assay, linked to herceptin, wins fda's okay. CAP Today.

[72] Monoclonal antibody approved for metastatic breast cancer (1998). Oncology (Williston Park).

[73] Barnes DM, Miles DW (2000). Response of metastatic breast cancer to trastuzumab?. Lancet.

[74] Mihaljevic A, Buchler P, Harder J, Hofheinz R, Gregor M, Kanzler S, Schmiegel W, Heinemann V, Endlicher E, Kloppel G, Seufferlein T, Geissler M (2009). A prospective, non-randomized phase-ii trial of trastuzumab and capecitabine in patients with her2 expressing advanced pancreatic cancer. BMC Surg.

[75] Santin AD, Bellone S, Roman JJ, McKenney JK, Pecorelli S (2008). Trastuzumab treatment in patients with advanced or recurrent endometrial carcinoma overexpressing her2/neu. Int J Gynaecol Obstet.

[76] Liang CH, Shiu LY, Chang LC, Sheu HM, Tsai EM, Kuo KW (2008). Solamargine enhances her2 expression and increases the susceptibility of human lung cancer h661 and h69 cells to trastuzumab and epirubicin. Chem Res Toxicol.

[77] Chavez-Blanco A, Perez-Sanchez V, Gonzalez-Fierro A, Vela-Chavez T, Candelaria M, Cetina L, Vidal S, Duenas-Gonzalez A (2004). Her2 expression in cervical cancer as a potential therapeutic target. BMC Cancer.

[78] Serrano-Olvera A, Duenas-Gonzalez A, Gallardo-Rincon D, Candelaria M, De la Garza-Salazar J (2006). Prognostic, predictive and therapeutic implications of her2 in invasive epithelial ovarian cancer. Cancer Treat Rev.

[79] Cohen MH, Johnson JR, Chen YF, Sridhara R, Pazdur R (2005). Fda drug approval summary: Erlotinib (tarceva) tablets. Oncologist.

[80] Erlotinib: Cp 358774, nsc 718781, osi 774, r 1415 (2003). Drugs R D.

[81] Herbst RS, Sandler A (2008). Bevacizumab and erlotinib: A promising new approach to the treatment of advanced nsclc. Oncologist.

[82] Senderowicz AM, Johnson JR, Sridhara R, Zimmerman P, Justice R, Pazdur R (2007). Erlotinib/gemcitabine for first-line treatment of locally advanced or metastatic adenocarcinoma of the pancreas. Oncology (Williston Park).

[83] Saif MW (2008). Erlotinib: The first biologic in the management of pancreatic cancer. Expert Opin Pharmacother.

[84] Dickler MN, Rugo HS, Eberle CA, Brogi E, Caravelli JF, Panageas KS, Boyd J, Yeh B, Lake DE, Dang CT, Gilewski TA, Bromberg JF, Seidman AD, D'Andrea GM, Moasser MM, Melisko M, Park JW, Dancey J, Norton L, Hudis CA (2008). A phase ii trial of erlotinib in combination with bevacizumab in patients with metastatic breast cancer. Clin Cancer Res.

[85] Thomas MB, Morris JS, Chadha R, Iwasaki M, Kaur H, Lin E, Kaseb A, Glover K, Davila M, Abbruzzese J (2009). Phase ii trial of the combination of bevacizumab and erlotinib in patients who have advanced hepatocellular carcinoma. J Clin Oncol.

[86] Bukowski RM, Kabbinavar FF, Figlin RA, Flaherty K, Srinivas S, Vaishampayan U, Drabkin HA, Dutcher J, Ryba S, Xia Q, Scappaticci FA, McDermott D (2007). Randomized phase ii study of erlotinib combined with bevacizumab compared with bevacizumab alone in metastatic renal cell cancer. J Clin Oncol.

[87] Van Cutsem E, Verslype C, Beale P, Clarke S, Bugat R, Rakhit A, Fettner SH, Brennscheidt U, Feyereislova A, Delord JP (2008). A phase ib dose-escalation study of erlotinib, capecitabine and oxaliplatin in metastatic colorectal cancer patients. Ann Oncol.

[88] Gravis G, Bladou F, Salem N, Goncalves A, Esterni B, Walz J, Bagattini S, Marcy M, Brunelle S, Viens P (2008). Results from a monocentric phase ii trial of erlotinib in patients with metastatic prostate cancer. Ann Oncol.

[89] Nogueira-Rodrigues A, do Carmo CC, Viegas C, Erlich F, Camisao C, Fontao K, Lima R, Herchenhorn D, Martins RG, Moralez GM, Small IA, Ferreira CG (2008). Phase i trial of erlotinib combined with cisplatin and radiotherapy for patients with locally advanced cervical squamous cell cancer. Clin Cancer Res.

[90] Wilhelm S, Carter C, Lynch M, Lowinger T, Dumas J, Smith RA, Schwartz B, Simantov R, Kelley S (2006). Discovery and development of sorafenib: A multikinase inhibitor for treating cancer. Nat Rev Drug Discov.

[91] Kane RC, Farrell AT, Madabushi R, Booth B, Chattopadhyay S, Sridhara R, Justice R, Pazdur R (2009). Sorafenib for the treatment of unresectable hepatocellular carcinoma. Oncologist.

[92] Kane RC, Farrell AT, Saber H, Tang S, Williams G, Jee JM, Liang C, Booth B, Chidambaram N, Morse D, Sridhara R, Garvey P, Justice R, Pazdur R (2006). Sorafenib for the treatment of advanced renal cell carcinoma. Clin Cancer Res.

[93] Chowdhury S, Larkin JM, Gore ME (2008). Recent advances in the treatment of renal cell carcinoma and the role of targeted therapies. Eur J Cancer.

[94] Izzedine H, Buhaescu I, Rixe O, Deray G (2007). Sunitinib malate. Cancer Chemother Pharmacol.

[95] Rock EP, Goodman V, Jiang JX, Mahjoob K, Verbois SL, Morse D, Dagher R, Justice R, Pazdur R (2007). Food and drug administration drug approval summary: Sunitinib malate for the treatment of gastrointestinal stromal tumor and advanced renal cell carcinoma. Oncologist.

[96] Goodman VL, Rock EP, Dagher R, Ramchandani RP, Abraham S, Gobburu JV, Booth BP, Verbois SL, Morse DE, Liang CY, Chidambaram N, Jiang JX, Tang S, Mahjoob K, Justice R, Pazdur R (2007). Approval summary: Sunitinib for the treatment of imatinib refractory or intolerant gastrointestinal stromal tumors and advanced renal cell carcinoma. Clin Cancer Res.

[97] Zhu AX, Raymond E (2009). Early development of sunitinib in hepatocellular carcinoma. Expert Rev Anticancer Ther.

[98] Taran A, Ignatov A, Smith B, Costa SD, Bischoff J (2008). Acute hepatic failure following monotherapy with sunitinib for ovarian cancer. Cancer Chemother Pharmacol.

[99] Park IH, Kwon Y, Kim EA, Lee KS, Ro J (2008). Major response to sunitinib (sutene(r)) in metastatic malignant phyllodes tumor of breast. Invest New Drugs.

[100] Socinski MA (2008). The current status and evolving role of sunitinib in non-small cell lung cancer. J Thorac Oncol.

[101] Rini BI (2007). Sunitinib. Expert Opin Pharmacother.

[102] Gao N, Zhang Z, Jiang BH, Shi X (2003). Role of pi3k/akt/mtor signaling in the cell cycle progression of human prostate cancer. Biochem Biophys Res Commun.

[103] Dormond O, Madsen JC, Briscoe DM (2007). The effects of mtor-akt interactions on anti-apoptotic signaling in vascular endothelial cells. J Biol Chem.

[104] Giles FJ, Albitar M (2005). Mammalian target of rapamycin as a therapeutic target in leukemia. Curr Mol Med.

[105] Martelli AM, Tazzari PL, Evangelisti C, Chiarini F, Blalock WL, Billi AM, Manzoli L, McCubrey JA, Cocco L (2007). Targeting the phosphatidylinositol 3-kinase/akt/mammalian target of rapamycin module for acute myelogenous leukemia therapy: From bench to bedside. Curr Med Chem.

[106] Temsirolimus: Cci 779, cci-779, cell cycle inhibitor-779 (2004). Drugs R D.

[107] Sadler TM, Gavriil M, Annable T, Frost P, Greenberger LM, Zhang Y (2006). Combination therapy for treating breast cancer using antiestrogen, era-923, and the mammalian target of rapamycin inhibitor, temsirolimus. Endocr Relat Cancer.

[108] Thallinger C, Poeppl W, Pratscher B, Mayerhofer M, Valent P, Tappeiner G, Joukhadar C (2007). Cci-779 plus cisplatin is highly effective against human melanoma in a scid mouse xenotranplantation model. Pharmacology.

[109] Thallinger C, Werzowa J, Poeppl W, Kovar FM, Pratscher B, Valent P, Quehenberger P, Joukhadar C (2007). Comparison of a treatment strategy combining cci-779 plus dtic versus dtic monotreatment in human melanoma in scid mice. J Invest Dermatol.

[110] Figlin RA, Brown E, Armstrong AJ, Akerley W, Benson AB, Burstein HJ, Ettinger DS, Febbo PG, Fury MG, Hudes GR, Kies MS, Kwak EL, Morgan RJ, Mortimer J, Reckamp K, Venook AP, Worden F, Yen Y (2008). Nccn task force report: Mtor inhibition in solid tumors. J Natl Compr Canc Netw.

[111] Garcia JA, Danielpour D (2008). Mammalian target of rapamycin inhibition as a therapeutic strategy in the management of urologic malignancies. Mol Cancer Ther.

[112] Sillaber C, Mayerhofer M, Bohm A, Vales A, Gruze A, Aichberger KJ, Esterbauer H, Pfeilstocker M, Sperr WR, Pickl WF, Haas OA, Valent P (2008). Evaluation of antileukaemic effects of rapamycin in patients with imatinib-resistant chronic myeloid leukaemia. Eur J Clin Invest.

[113] Johnston SR, Martin LA, Leary A, Head J, Dowsett M (2007). Clinical strategies for rationale combinations of aromatase inhibitors with novel therapies for breast cancer. J Steroid Biochem Mol Biol.

[114] Chollet P, Abrial C, Tacca O, Mouret-Reynier MA, Leheurteur M, Durando X, Cure H (2006). Mammalian target of rapamycin inhibitors in combination with letrozole in breast cancer. Clin Breast Cancer.

[115] Recher C, Dos Santos C, Demur C, Payrastre B (2005). Mtor, a new therapeutic target in acute myeloid leukemia. Cell Cycle.

[116] Rizzieri DA, Feldman E, Dipersio JF, Gabrail N, Stock W, Strair R, Rivera VM, Albitar M, Bedrosian CL, Giles FJ (2008). A phase 2 clinical trial of deforolimus (ap23573, mk-8669), a novel mammalian target of rapamycin inhibitor, in patients with relapsed or refractory hematologic malignancies. Clin Cancer Res.

[117] Roccaro AM, Hideshima T, Richardson PG, Russo D, Ribatti D, Vacca A, Dammacco F, Anderson KC (2006). Bortezomib as an antitumor agent. Curr Pharm Biotechnol.

[118] Fernandez Y, Verhaegen M, Miller TP, Rush JL, Steiner P, Opipari AW, Lowe SW, Soengas MS (2005). Differential regulation of noxa in normal melanocytes and melanoma cells by proteasome inhibition: Therapeutic implications. Cancer Res.

[119] Musto P, Falcone A, Sanpaolo G, Guglielmelli T, Zambello R, Balleari E, Catalano L, Spriano M, Cavallo F, La Sala A, Mantuano S, Nobile M, Melillo L, Scalzulli PR, Dell'Olio M, Bodenizza C, Greco MM, Carella AM, Merla E, Carella AM, Boccadoro M, Cascavilla N, Palumbo A (2006). Bortezomib (velcade) for progressive myeloma after autologous stem cell transplantation and thalidomide. Leuk Res.

[120] Kane RC, Farrell AT, Sridhara R, Pazdur R (2006). United states food and drug administration approval summary: Bortezomib for the treatment of progressive multiple myeloma after one prior therapy. Clin Cancer Res.

[121] Horwitz SM (2008). Novel therapies for cutaneous t-cell lymphomas. Clin Lymphoma Myeloma.

[122] Zinzani PL, Musuraca G, Tani M, Stefoni V, Marchi E, Fina M, Pellegrini C, Alinari L, Derenzini E, de Vivo A, Sabattini E, Pileri S, Baccarani M (2007). Phase ii trial of proteasome inhibitor bortezomib in patients with relapsed or refractory cutaneous t-cell lymphoma. J Clin Oncol.

[123] Younes A, Pro B, Fayad L (2006). Experience with bortezomib for the treatment of patients with relapsed classical hodgkin lymphoma. Blood.

[124] Abayomi EA, Sissolak G, Jacobs P (2007). Use of novel proteosome inhibitors as a therapeutic strategy in lymphomas current experience and emerging concepts. Transfus Apher Sci.

[125] Anargyrou K, Dimopoulos MA, Sezer O, Terpos E (2008). Novel anti-myeloma agents and angiogenesis. Leuk Lymphoma.

[126] Barlogie B, Desikan R, Eddlemon P, Spencer T, Zeldis J, Munshi N, Badros A, Zangari M, Anaissie E, Epstein J, Shaughnessy J, Ayers D, Spoon D, Tricot G (2001). Extended survival in advanced and refractory multiple myeloma after single-agent thalidomide: Identification of prognostic factors in a phase 2 study of 169 patients. Blood.

[127] Rajkumar SV, Blood E, Vesole D, Fonseca R, Greipp PR (2006). Phase iii clinical trial of thalidomide plus dexamethasone compared with dexamethasone alone in newly diagnosed multiple myeloma: A clinical trial coordinated by the eastern cooperative oncology group. J Clin Oncol.

[128] Rajkumar SV, Rosinol L, Hussein M, Catalano J, Jedrzejczak W, Lucy L, Olesnyckyj M, Yu Z, Knight R, Zeldis JB, Blade J (2008). Multicenter, randomized, double-blind, placebo-controlled study of thalidomide plus dexamethasone compared with dexamethasone as initial therapy for newly diagnosed multiple myeloma. J Clin Oncol.

[129] Pinter M, Wichlas M, Schmid K, Plank C, Muller C, Wrba F, Peck-Radosavljevic M (2008). Thalidomide in advanced hepatocellular carcinoma as antiangiogenic treatment approach: A phase i/ii trial. Eur J Gastroenterol Hepatol.

[130] Knisely JP, Berkey B, Chakravarti A, Yung AW, Curran WJ, Robins HI, Movsas B, Brachman DG, Henderson RH, Mehta MP (2008). A phase iii study of conventional radiation therapy plus thalidomide versus conventional radiation therapy for multiple brain metastases (rtog 0118). Int J Radiat Oncol Biol Phys.

[131] Barr P, Fu P, Lazarus H, Kane D, Meyerson H, Hartman P, Reyes R, Creger R, Stear K, Laughlin M, Tse W, Cooper B (2007). Antiangiogenic activity of thalidomide in combination with fludarabine, carboplatin, and topotecan for high-risk acute myelogenous leukemia. Leuk Lymphoma.

[132] McMeekin DS, Sill MW, Darcy KM, Stearns-Kurosawa DJ, Webster K, Waggoner S, Benbrook D (2007). A phase ii trial of thalidomide in patients with refractory leiomyosarcoma of the uterus and correlation with biomarkers of angiogenesis: A gynecologic oncology group study. Gynecol Oncol.

[133] Bujak A, Kalas W (2008). [metronomic chemotherapy: A new approach in cancer therapy]. Postepy Hig Med Dosw (Online).

[134] Correale P, Cerretani D, Remondo C, Martellucci I, Marsili S, La Placa M, Sciandivasci A, Paolelli L, Pascucci A, Rossi M, Di Bisceglie M, Giorgi G, Gotti G, Francini G (2006). A novel metronomic chemotherapy regimen of weekly platinum and daily oral etoposide in high-risk non-small cell lung cancer patients. Oncol Rep.

[135] Sanborn SL, Cooney MM, Dowlati A, Brell JM, Krishnamurthi S, Gibbons J, Bokar JA, Nock C, Ness A, Remick SC (2008). Phase i trial of docetaxel and thalidomide: A regimen based on metronomic therapeutic principles. Invest New Drugs.

[136] Stoelting S, Trefzer T, Kisro J, Steinke A, Wagner T, Peters SO (2008). Low-dose oral metronomic chemotherapy prevents mobilization of endothelial progenitor cells into the blood of cancer patients. In Vivo.

[137] Jurado JM, Sanchez A, Pajares B, Perez E, Alonso L, Alba E (2008). Combined oral cyclophosphamide and bevacizumab in heavily pre-treated ovarian cancer. Clin Transl Oncol.

[138] Dellapasqua S, Bertolini F, Bagnardi V, Campagnoli E, Scarano E, Torrisi R, Shaked Y, Mancuso P, Goldhirsch A, Rocca A, Pietri E, Colleoni M (2008). Metronomic cyclophosphamide and capecitabine combined with bevacizumab in advanced breast cancer. J Clin Oncol.

[139] Lopez-Aguilar E, Sepulveda-Vildosola AC, Betanzos-Cabrera Y, Rocha-Moreno YG, Gascon-Lastiri G, Rivera-Marquez H, Wanzke-del-Angel V, Cerecedo-Diaz F, de la Cruz-Yanez H (2008). Phase ii study of metronomic chemotherapy with thalidomide, carboplatin-vincristine-fluvastatin in the treatment of brain stem tumors in children. Arch Med Res.

[140] Sarmiento R, Gasparini G (2008). Antiangiogenic metronomic chemotherapy. Onkologie.

[141] Ghiringhelli F, Menard C, Puig PE, Ladoire S, Roux S, Martin F, Solary E, Le Cesne A, Zitvogel L, Chauffert B (2007). Metronomic cyclophosphamide regimen selectively depletes cd4+cd25+ regulatory t cells and restores t and nk effector functions in end stage cancer patients. Cancer Immunol Immunother.

[142] Generali D, Bates G, Berruti A, Brizzi MP, Campo L, Bonardi S, Bersiga A, Allevi G, Milani M, Aguggini S, Dogliotti L, Banham AH, Harris AL, Bottini A, Fox SB (2009). Immunomodulation of foxp3+ regulatory t cells by the aromatase inhibitor letrozole in breast cancer patients. Clin Cancer Res.

[143] Pasquier E, Kavallaris M, Andre N Metronomic chemotherapy: New rationale for new directions. Nat Rev Clin Oncol.

[144] Ma J, Waxman DJ (2009). Dominant effect of antiangiogenesis in combination therapy involving cyclophosphamide and axitinib. Clin Cancer Res.

[145] Blagosklonny MV (2004). Antiangiogenic therapy and tumor progression. Cancer Cell.

[146] Leroyer AS, Anfosso F, Lacroix R, Sabatier F, Simoncini S, Njock SM, Jourde N, Brunet P, Camoin-Jau L, Sampol J, Dignat-George F Endothelial-derived microparticles: Biological conveyors at the crossroad of inflammation, thrombosis and angiogenesis. Thromb Haemost.

[147] Carmeliet P, De Smet F, Loges S, Mazzone M (2009). Branching morphogenesis and antiangiogenesis candidates: Tip cells lead the way. Nat Rev Clin Oncol.

[148] Ruegg C, Alghisi GC Vascular integrins: Therapeutic and imaging targets of tumor angiogenesis. Recent Results Cancer Res.

[149] Sen CK, Gordillo GM, Khanna S, Roy S (2009). Micromanaging vascular biology: Tiny micrornas play big band. J Vasc Res.

[150] Khan N, Mukhtar H Cancer and metastasis: Prevention and treatment by green tea. Cancer Metastasis Rev.

[151] Banerjee S, Padhye S, Azmi A, Wang Z, Philip PA, Kucuk O, Sarkar FH, Mohammad RM Review on molecular and therapeutic potential of thymoquinone in cancer. Nutr Cancer.

